# JMM Profile: *Streptococcus pneumoniae:* sugar-coated captain of the men of death

**DOI:** 10.1099/jmm.0.001446

**Published:** 2021-11-15

**Authors:** Tina H. Dao, Jason W. Rosch

**Affiliations:** ^1^​ Department of Infectious Diseases, St. Jude Children’s Research Hospital, Memphis TN 38105, USA

**Keywords:** Pneumolysin, complement evasion, polysaccharide vaccines, Prevnar7, 13- PCV13, PPSV23, vaccine evasion, chloline requirement, optochin, bacitracin

## Abstract

*
Streptococcus pneumoniae* is a highly adept human pathogen. A frequent asymptomatic member of the respiratory microbiota, the pneumococcus has a remarkable capacity to cause mucosal (pneumonia and otitis media) and invasive diseases (bacteremia, meningitis). In addition, the organism utilizes a vast battery of virulence factors for tissue and immune evasion. Though recognized as a significant cause of pneumonia for over a century, efforts to develop more effective vaccines remain ongoing. The pathogen’s inherent capacity to exchange genetic material is critical to the pneumococcus’ success. This feature historically facilitated essential discoveries in genetics and is vital for disseminating antibiotic resistance and vaccine evasion.

## Historical perspective

*Streptococcus pneumoniae* was first isolated in 1881 and was established as the causative agent of pneumonia, the new ‘Captain of the men of death,’ shortly thereafter [1, 2]. Pneumococcal biology, specifically natural competence, facilitated seminal advances in genetics, including demonstrating the non-heritable exchange of genetic information and the role of DNA in transformation [3, 4].

## Clinical presentation

A frequent cause of acute otitis media, the pneumococcal middle ear infection is characterized by ear pain, swollen eardrum, fever and drowsiness. Pneumonia, the most common severe form of pneumococcal disease, presents with symptoms including fever and chills, cough, difficulty breathing and chest pain. Presentation of pneumococcal meningitis includes a stiff neck, fever, headache, confusion and sensitivity to light. Pneumococcal bacteremia is characterized by fever, chills, and low alertness and can progress to sepsis.

## Microbial characteristics: phenotypic and genotypic features

*S. pneumoniae* is a facultative anaerobe. The non-motile, non-spore-forming, alpha-haemolytic lancet-shaped diplococci or chains are resistant to bacitracin and sensitive to optochin and bile-mediated lysis. *S. pneumoniae* is genetically diverse with a core genome encoding between 500–1100 clusters of orthologues with a species-wide pan-genome predicted to encode up to >7000 orthologous genes [5]. Genetic variation amongst serotypes varies considerably. Some serotypes have relatively high genetic diversity, whereas others have a relatively homogeneous genetic background [6]. *S. pneumoniae* lacks genes required for the complete Entner–Doudoroff pathway and Krebs cycle. The pneumococci also have incomplete biosynthetic pathways for numerous amino acids, including cysteine, glycine, histidine, glutamine and glutamate [7]. *S. pneumoniae* primarily relies on carbohydrate fermentation coupled to substrate-level phosphorylation for energy generation [7].

## Clinical diagnosis, laboratory confirmation and safety

### Clinical diagnosis

Along with a constellation of clinically compatible signs and symptoms, including fever, cough, shortness of breath and rapid, shallow breathing, the diagnosis of pneumococcal pneumonia is supported by the radiographic demonstration of lobar consolidation with infiltrates in one or more segments within a single lobe on chest imaging. In addition, leukocytosis [white blood cells (WBC) >15 000 µl^−1^ blood] is usually associated with pneumococcal infection but may be low in severe cases.

### Laboratory confirmation

Pneumococcal infection confirmation is most often obtained by isolating *S. pneumoniae* from sputum, blood, respiratory secretions or throat swabs. To distinguish *S. pneumoniae* from bacteria of the normal nasopharyngeal flora, Gram-stained sputum from patients must contain predominantly Gram-positive diplococci with >25 white blood cells and <10 epithelial cells. *S. pneumoniae* can be isolated and cultured from the blood of patients with invasive infections in about 15 h. It is alpha-haemolytic, bacitracin-resistant and optochin-sensitive. In addition, it has a unique nutritional requirement, namely choline. Rapid diagnostic techniques include rapid point-of-care urine immunochromatographic membrane tests that detect pneumococcal C-polysaccharide antigen and quantitative PCR detecting the presence of the autolysin gene (*lytA*) from nasal or sputum samples. In addition, multilocus sequence typing (MLST) is frequently employed to identify clones associated with invasive infection [8].

### Laboratory safety

*S. pneumoniae* is classified as Biosafety Level-2 (BSL-2) by the Centers for Disease Control and Prevention (CDC) and Hazard Level 2 pathogen by the Advisory Committee on Dangerous Pathogens. It is also listed as medium priority pathogen by the World Health Organization (WHO). Thus, appropriate precautions should be taken when handling *S. pneumoniae *.

## Treatment and resistance

### Treatment

For mild pneumococcal infections, amoxicillin, second- or third-generation cephalosporins, or oral levofloxacin, are recommended [9]. Amoxicillin is the first-line therapy for healthy children for mild to moderate paediatric pneumococcal pneumonia caused by penicillin-sensitive strains. Alternatives include third-generation cephalosporins, including ceftriaxone or cefotaxime. For community-acquired pneumonia in adults, macrolides (azithromycin, erythromycin or clarithromycin) or a respiratory fluoroquinolone (moxifloxacin, gemifloxacin or levofloxacin) are recommended for mild cases with susceptible organisms. In severe cases, ceftriaxone or cefotaxime are first-line agents, followed by vancomycin for life-threatening infection [10]. Ceftriaxone is the first line of therapy to treat pneumococcal meningitis caused by susceptible strains.

### Resistance

Approximately 30 % of clinical strains harbour resistance to at least one antibiotic, and many strains carry multidrug resistance [11]. Resistance to β-lactam antibiotics requires recombination events between related streptococcal species resulting in mosaic penicillin-binding proteins, PBP2b, PBP2x and PBP1a, with decreased affinity for β-lactams [12]. Macrolide resistance increased strikingly in recent years, partially due to the reduced frequency of penicillin-resistant strains targeted by vaccines [13] and the widespread use of macrolide. The macrolide resistance is due to ribosome demethylation to prevent antibiotic binding to 23S rRNA and acquisition of macrolide efflux (Mef) pumps [14]. Trimethoprim-sulfamethoxazole resistance rates can be >60 % and are often associated with other antibiotic resistances [13]. Fitness tradeoffs related to the acquisition of resistance coupled with selective targeting of serotypes by vaccination are important determinants dictating the prevalence of resistance.

## Pathogenic strategies

### Host range

The only known natural host for *S. pneumoniae* is humans.

### Transmission

*S. pneumoniae* is transmitted through inhalation of infectious aerosols or direct contact with infected mucous membranes or secretions. In addition, influenza co-infection can enhance transmission by both contact-dependent and airborne routes [15, 16].

### Infection

A typical commensal respiratory flora, the pneumococcus can invade and infect numerous organs, including the middle ear, lungs, bloodstream, heart and brain, each with distinctive disease manifestations.

### Host response and evasion

The host response to pneumococcal pneumonia initially involves clearance by alveolar macrophages following rapid and intense neutrophil infiltration. *S. pneumoniae* polysaccharide capsules, of which 100 have been identified, confer resistance to phagocytosis-mediated clearance. In addition, the high selective pressure imparted by vaccination can result in capsular switching to evade vaccines [17]. The pneumococcus can also evade mucosal antibody defenses via IgA1 metalloprotease, which cleaves the mucosal IgA [18].

### Virulence factors

**Fig. 1.**
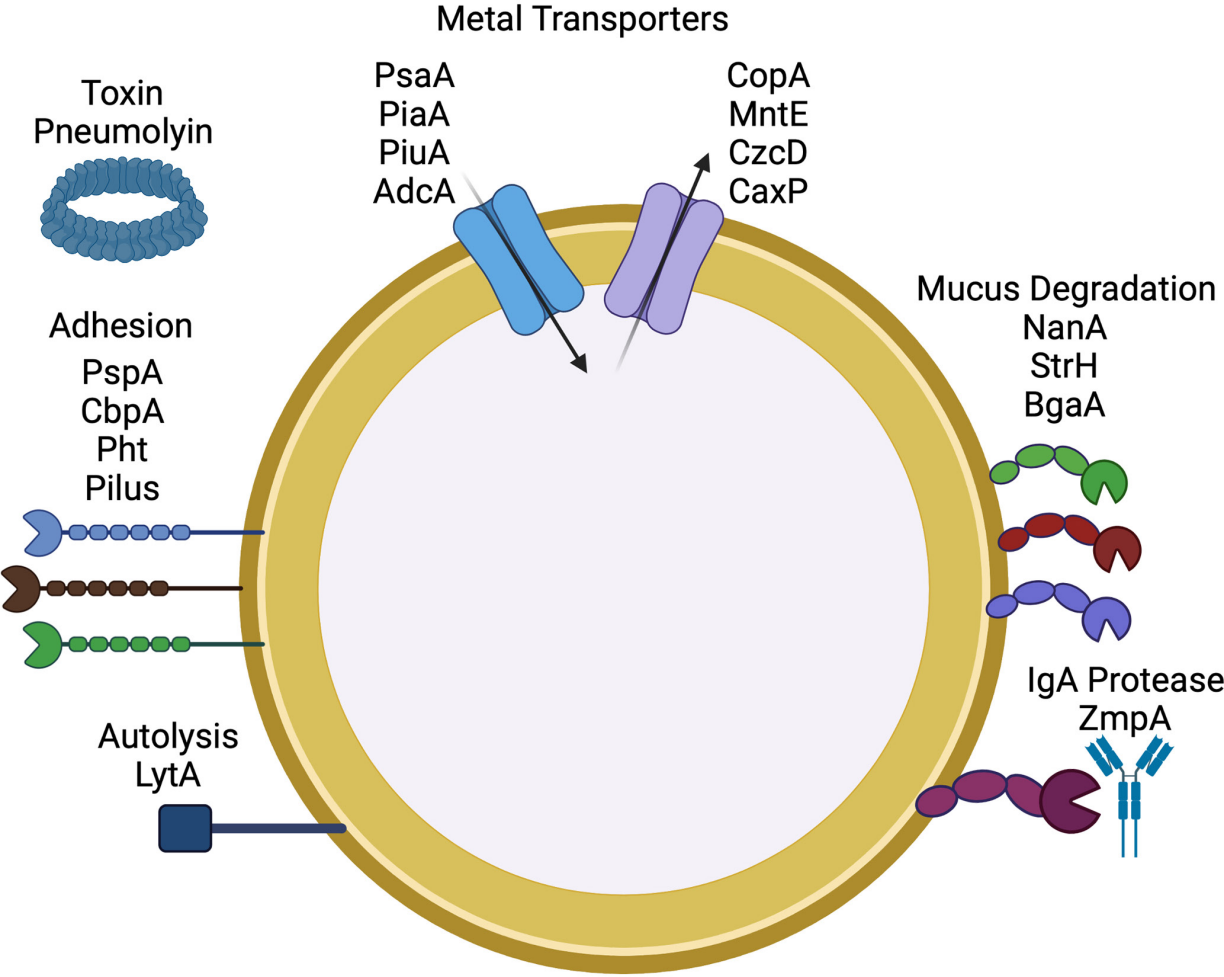
Pneumococcal virulence factors. Critical to the success of *S. pneumoniae* is the capacity to survive within the human host. This encompasses mechanisms required for nutrient acquisition such as metal ions and carbohydrate sources, adhesion and evasion of host defenses.

Critical to the invasive capacity of the pneumococcus is its ability to adhere to and damage host cells and evade innate immunity using a multitude of mechanisms (Fig. 1) [19]. One major virulence factor is the capsule. The pneumococcus contains phosphorylcholine on both the cell-wall teichoic acid and the membrane-associated lipoteichoic acid [20]. The choline-binding proteins (CBPs) have been implicated in several cellular processes, including autolysis, adhesion and host-cell invasion [21]. *S. pneumoniae* is extremely successful in circumventing the complement activation pathways by preventing opsonization (capsule, PspA, LytA), depleting complement components (Ply, PepO, PGK, glyceraldehyde-3-phosphate dehydrogenase), inhibiting C3 convertase formation (Eno, CbpA, PhtS, LytA, PepP) and MAC assembly (CbpA and phosphoglycerate kinase) [22]. The primary toxin, pneumolysin (PLY protein) produced by all strains, is a cholesterol-dependent cytolysin cytotoxic to most host cells and interferes with host responses by modulating host complement pathways allowing *S. penumoniae* to evade and thrive. PLY induces apoptosis of macrophages and brain cells, activates the production of inflammatory cytokines, activates NLRP3 inflammasome (implicated in neuroinflammation), induces neutrophil extracellular trap (NET) formation, among others [23].

## Epidemiology, prevention and risk groups

### Epidemiology

Pneumococcal colonization and infection are endemic in all geographic regions and are the leading cause of pneumonia mortality worldwide [24]. Globally, it is estimated half-a-million children in the developing world are killed annually by *S. pneumoniae *. Invasive disease, whereby bacteria are recovered from normally sterile sites, predominantly impacts the young (<2 years) and elderly (>65 years). Following the introduction of the pneumococcal conjugate vaccine (Prevnar-7), a near-complete replacement of non-vaccine types was observed during carriage and reduced the incidence of invasive infections [25].

### Prevention

The three pneumococcal vaccines: conjugate 7-valent (PCV7), 13-valent (PCV13) and pneumococcal polysaccharide (PPSV23) vaccines, are made of 7, 13 and 23 polysaccharide types, respectively. PCV77 and PCV13 are recommended for those younger than 2 years and those with certain medical conditions. The PPSV23 is recommended for adults older than 65 years, individuals with specific genetic (sickle cell disease) and medical conditions (impaired splenic function), and smokers are advised to vaccinate with PPSV23 to prevent invasive pneumococcal infection (https://www.cdc.gov/pneumococcal/vaccination.html). In addition, patients with sickle cell disease, who are highly susceptible to invasive pneumococcal disease, are recommended to receive penicillin prophylaxis for the first 5 years of life in addition to vaccination to reduce the risk of invasive infection further [26].

### Risk groups

Besides age, other risk factors for the invasive pneumococcal disease include alcohol abuse, smoking, chronic lung disease, diabetes and asthma [27]. Prior viral co-infection, particularly with influenza virus, can exacerbate secondary bacterial pneumonia caused by *S. pneumoniae* and significantly contribute to morbidity and mortality in influenza pandemics. Individuals with genetic (sickle cell and complement deficiency) and medical (asplenic and immunocompromised) deficiencies and individuals with solid or haematologic malignancies are also at high risk of invasive pneumococcal disease [27].

## Open questions

- What are the optimal candidates for vaccine development to prevent pneumonia and other infections at the mucosal surface?
- How will the antibiotic resistance, particularly multidrug resistance, of circulating strains change over the next 10 years?
- How do determinants of pathogenicity in this genetically diverse pathogen vary amongst clinical isolates?
